# AAV-Mediated Artificial miRNA Reduces Pathogenic Polyglucosan Bodies and Neuroinflammation in Adult Polyglucosan Body and Lafora Disease Mouse Models

**DOI:** 10.1007/s13311-022-01218-7

**Published:** 2022-03-28

**Authors:** Emrah Gumusgoz, Sahba Kasiri, Dikran R. Guisso, Jun Wu, Matthew Dear, Brandy Verhalen, Berge A. Minassian

**Affiliations:** 1 Division of Neurology, Department of Pediatrics, University of Texas Southwestern Medical Center, 5323 Harry Hines Boulevard, Dallas, TX 75390 USA; 2 Present affiliation: Corteva Agriscience, Johnston, IA 50131 USA

**Keywords:** EPM2A, EPM2B, GYS1, GBE1, APBD, RNAi, miRNA, AAV9

## Abstract

Adult polyglucosan body disease (APBD) and Lafora disease (LD) are autosomal recessive glycogen storage neurological disorders. APBD is caused by mutations in the glycogen branching enzyme (*GBE1*) gene and is characterized by progressive upper and lower motor neuron dysfunction and premature death. LD is a fatal progressive myoclonus epilepsy caused by loss of function mutations in the *EPM2A* or *EPM2B* gene. These clinically distinct neurogenetic diseases share a common pathology. This consists of time-dependent formation, precipitation, and accumulation of an abnormal form of glycogen (polyglucosan) into gradually enlarging inclusions, polyglucosan bodies (PBs) in ever-increasing numbers of neurons and astrocytes. The growth and spread of PBs are followed by astrogliosis, microgliosis, and neurodegeneration. The key defect in polyglucosans is that their glucan branches are longer than those of normal glycogen, which prevents them from remaining in solution. Since the lengths of glycogen branches are determined by the enzyme glycogen synthase, we hypothesized that downregulating this enzyme could prevent or hinder the generation of the pathogenic PBs. Here, we pursued an adeno-associated virus vector (AAV) mediated RNA-interference (RNAi) strategy. This approach resulted in approximately 15% reduction of glycogen synthase mRNA and an approximately 40% reduction of PBs across the brain in the APBD and both LD mouse models. This was accompanied by improvements in early neuroinflammatory markers of disease. This work represents proof of principle toward developing a single lifetime dose therapy for two fatal neurological diseases: APBD and LD. The approach is likely applicable to other severe and common diseases of glycogen storage.

**Supplementary Information:**

The online version contains supplementary material available at 10.1007/s13311-022-01218-7.

## Introduction

Adult polyglucosan body disease (APBD) is a fatal adult-onset neurodegenerative disorder characterized by progressive sensory deficits, upper and lower motor neuron signs, ataxia, and loss of urinary bladder control. It is caused by mutations in the *GBE1* gene, which encodes the glycogen branching enzyme (GBE) [1, 2]. This is one of the two main enzymes that synthesize glycogen, the other being glycogen synthase. As soon as glycogen synthase elongates a glycogen branch, GBE cleaves this extension, en bloc, and reattaches it upstream, converting the linear glucan to a fork. Glycogen synthase elongates each prong of the fork, which GBE branches, and so forth. The final macromolecule, the largest molecule in the cytosol, occupies a spherical space with all the branches pointing away from each other, allowing it to be perfused with water and thus soluble [3]. In APBD, GBE cannot keep pace with glycogen synthase, and glucan chains become too long. Glycogen with overlong branches (termed polyglucosan) precipitates, possibly because long branches wind around each other and extrude water, as in plant starches [4]. Polyglucosans aggregate, and accumulate into polyglucosan bodies (PBs), which provoke neuroinflammation and neurodegeneration [1, 2, 5]. PBs form in glia and neurons, in the latter in axons, which they appear to clog, perhaps explaining the disease’s axonopathic neurological presentation [6].

Lafora disease (LD) is a fatal teenage-onset progressive myoclonus epilepsy. It is caused by mutations in the *EPM2A* or *EPM2B* genes, which encode the laforin glycogen phosphatase and its interacting malin E3 ubiquitin ligase [7, 8]. The precise functions of laforin and malin are not known—although glycogen synthase regulation is a distinct possibility—but a loss of either leads to overlong glycogen branches and profuse PB formation very similar to APBD, minus the peculiar axonal localization. As in APBD, there is neuroinflammation and neurodegeneration [9–13]. Why LD is characterized by a severe epilepsy is unknown, but may in part relate to preferential loss of GABAergic inhibitory interneurons [14, 15].

Since overextended glycogen branches appear to be a pathogenic nexus in APBD and LD, it was reasoned that downregulating glycogen synthase may be therapeutic. Glycogen synthase was downregulated in the mouse models of APBD and LD by (1) germline or postnatal transgenic knockout or knockdown of the glycogen synthase gene *Gys1* or of genes (*Ppp1r3c*, *Ppp1r3d*) that encode glycogen synthase activating enzymes [16–23], (2) postnatal *Gys1* knockout with virally delivered CRISPR/Cas9 [5], and (3) postnatal *Gys1* knockdown with an antisense oligonucleotide (ASO) [24]. In all cases, PB formation was prevented or halted, neuropathological features corrected, and, where tested, behavioral, motor, or epileptic abnormalities rescued [5, 16–24]. Here, toward developing a lifetime single-dose therapy for APBD and LD, we deliver a *Gys1*-targeting artificial microRNA (amiRNA) via AAV9 to their corresponding mouse models and demonstrate major reductions in PB formation and ameliorations of disease neuroinflammatory markers.

## Methods

### Study Design

The selected *Gys1*-targeting AAV-amiRNA, or phosphate-buffered saline (PBS), was administered at postnatal day 2 by intracerebroventricular (ICV) injection. Mice were sacrificed at 3 months of age, and the effects of the treatment on PB quantity and neuroinflammatory markers were analyzed.

### Plasmid Construction and Viral Packaging

**Fig. 1:**
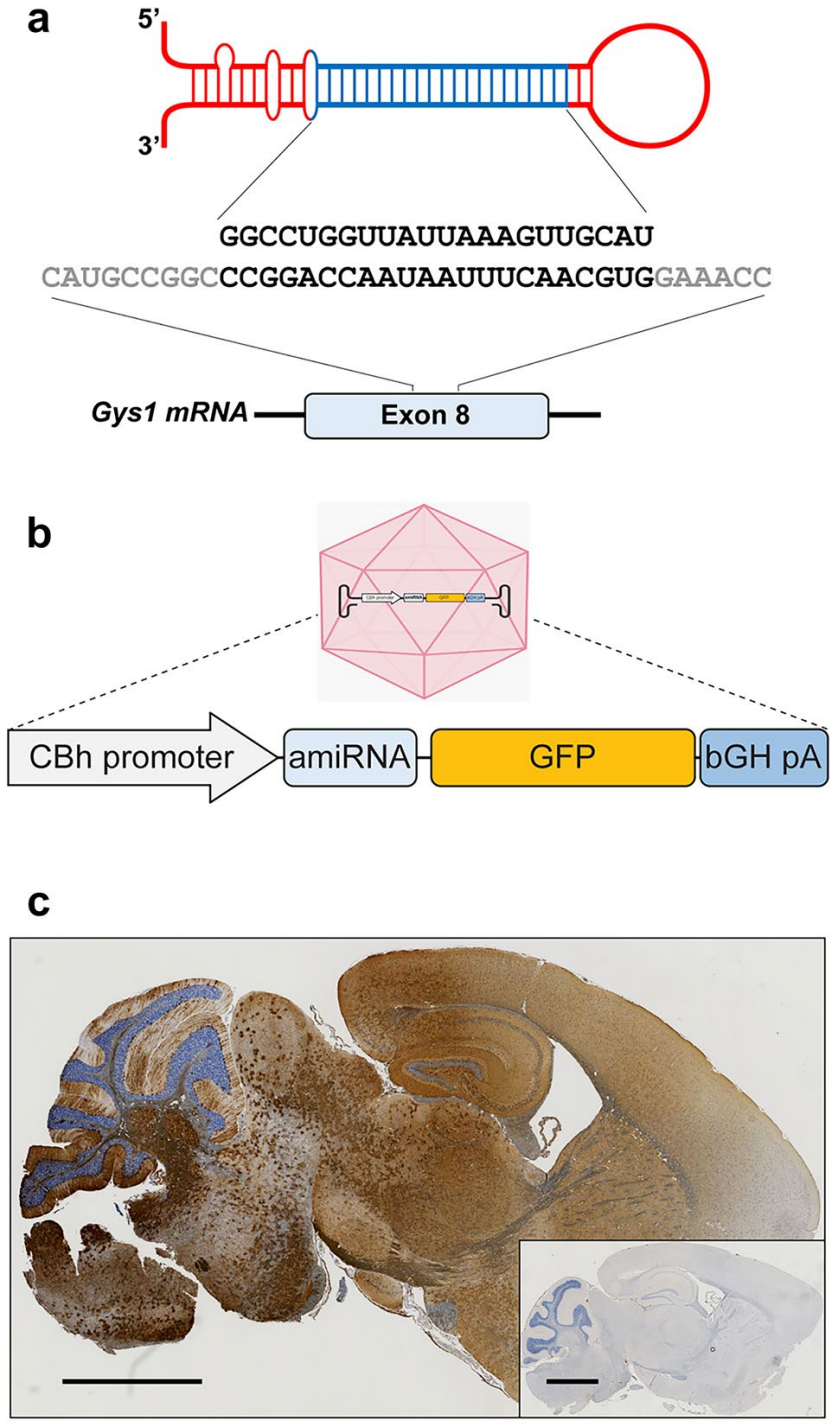
AAV-amiRNA targeting murine *Gys1* mRNA. Schematic representation of AAV-amiRNA and target site sequences (**a**). AAV-amiRNA vector components: CBh, hybrid chicken beta-actin promoter; GFP, enhanced green fluorescent protein; bGH-pA, bovine growth hormone polyadenylation signal (**b**). Representative images show GFP expression in sagittal brain sections of AAV-amiRNA treated (**c**) vs PBS-treated mice (inset of **c**). Scale bar, 2 mm

*Gys1* targeting siRNA sequences were predicted using siSPOTR [25]. Using Gibson Assembly (NEB), these were cloned into the natural human/mouse miR-30 pri-miRNA scaffold [26] and a green fluorescent protein (GFP) coding sequence containing AAV-based expression vector [27] (Fig. 1a, b). The CBh promoter was used to drive the expression of the amiRNA and GFP simultaneously. A 3’ bGH poly(A) signal stabilized the transcript (Fig. 1b). The cassette was flanked by AAV2 inverted terminal repeats and cloned in a self-complementary fashion for enhanced post-transduction stability and expression. The final plasmid was packaged in AAV9 at the University of North Carolina Vector Core facility as described [28].

### Mice

APBD (*Gbe1*^*Y239S*^) and LD (*Epm2a*^*−/−*^ and *Epm2b*^*−/−*^) mouse models were described previously [29–31]. Both sexes were used in approximately equal proportion in all experiments. All procedures were carried out according to NIH guidelines and the Institutional Animal Care and Use Committee regulations at the University of Texas Southwestern Medical Center. 5 × 10^11^ vector genomes (in 5 μl solution) or 5 μl PBS were injected ICV as described [32]. Mice were sacrificed by cervical dislocation at 3 months, and the brain was harvested and cut into two hemispheres. One hemisphere was fixed in formalin for paraffin embedding and histo- and immunohistochemistry analyses, and the other snap-frozen in liquid nitrogen, later ground into powder by mortar and pestle. After being ground into powder, the tissue was aliquoted into 30–40 mg powder in screw cap tubes for the genetic and biochemical experiments below.

### GFP Immunohistochemistry

Paraffin-embedded tissues were sectioned at the UT Southwestern Medical Center HistoPathology Core, Sects. (5 μm) were mounted on glass slides, de-paraffinized and rehydrated by processing through xylene and decreasing concentrations of ethanol in water and subjected to antigen retrieval using citrate buffer pH 6.0 (Sigma). Endogenous peroxidase activity was blocked for 10 min with BLOXALL solution (Vector Labs). Sections were incubated with rabbit anti-GFP antibody (1:50, Santa Cruz) diluted in normal horse serum overnight at 4 °C, then successively incubated with Amplifier Antibody and ImmPRESS Polymer Reagent (Vector Labs) and the ImmPACT DAB EqV working solution (Vector Labs) until desired stain intensity.

### *Gys1* mRNA Extraction and Quantification by Droplet Digital PCR

RNA was extracted using TriZol (Invitrogen) and purified using PureLink RNA Mini Kit (Invitrogen) following manufacturer instructions. cDNA was generated using the iScript Reverse Transcription SuperMix kit (Bio-Rad Laboratories). *Gys1* RNA was quantified using the Bio-Rad QX200 Droplet Digital PCR (ddPCR) system as instructed by the manufacturer. *Tfrc* was used as a reference gene. Custom-designed TaqMan primers and probes (Thermo Fisher Scientific) were used for both *Gys1* and *Tfrc*. ddPCR reactions were assembled using standard protocols. The 20 μL reaction mix consisted of 10 μL 2 × ddPCR SuperMix for Probes (Bio-Rad Laboratories), 1 μL *Gys1* assay mix (FAM-labeled), 1 μL reference *Tfrc* assay mix (VIC-labeled), 3 μL of cDNA, and 5 μL nuclease-free water. Cycling conditions were 95 °C for 10 min, 45 cycles of 94 °C for 30 s and 60 °C for 1 min, and 98 °C for 10 min. The resulting data was quantified by QuantaSoft v1.4 (Bio-Rad). Results were expressed as a ratio of *Gys1*/*Tfrc*. No-template controls were run in parallel with study samples. Primer/probe sets are listed in Table 1.

**Table 1:** Primer and probe sequences used in this study

Gene	Forward primer (5′ 3’)	Reverse primer (5′ 3’)
*Gys1 (ddPCR)*	CAGAGCAAAGCACGAATCCA	CATAGCGGCCAGCGATAAAG
*Gys1 Probe (ddPCR)*	TTATGGGCACCTGGAC	
*RTfrc (ddPCR)*	TGCCTAATATACCTGTGCAAACAATC	TTCCTTCCATTTTTCCAAATAGCT
*Tfrc Probe (ddPCR)*	CAAGAGCTGCTGCAGAA	
*Rpl4*	CCCTTACGCCAAGACTATGC	TGGAACAACCTTCTCGGATT
*Cxcl10*	AAGTGCTGCCGTCATTTTCT	ATAGGCTCGCAGGGATGATT
*Ccl5*	TGCCAACCCAGAGAAGAAGT	AGCAAGCAATGACAGGGAAG
*Lcn2*	GCCTCAAGGACGACAACATC	CACACTCACCACCCATTCAG
*C3*	CTGTGTGGGTGGATGTGAAG	TCCTGAGTGTCGTTTGTTGC

### GFP and GYS1 Western Blots

Tissue lysate was obtained by homogenizing frozen ground brain tissue using ice-cold RIPA lysis buffer (150 mM NaCl, 1% NP-40, 0.5% sodium deoxycholate, 0.1% sodium dodecyl sulfate, 50 mM Tris–HCl pH 8.0) containing protease inhibitors (1 mM PMSF, 5 μg/mL Leupetin, 10 μg/mL Pepstatin, 20 KIU/mL Aprotinin, 50 mM NaF). Lysates were centrifuged at 14,000 × *g* for 5 min at 4 °C and supernatants were collected. Protein concentration was measured using the Bradford assay reagent (ThermoScientific). The standard curve for protein quantification was generated using a serial dilution of albumin standard (ThermoScientific). Equal amounts of whole protein from each sample were subjected to SDS-PAGE using the TGX Stain-Free FastCast Acrylamide kit (Bio-Rad). Protein bands were transferred to polyvinylidene difluoride (PVDF) membrane (Millipore) overnight at 4 °C. Primary antibodies for GFP (1:1000, Santa Cruz) and for GYS1 (1:1000, Cell Signalling) were used. Protein densitometry was performed using Image Lab software (Bio-Rad Laboratories). The intensity of each protein band was normalized to the intensity of its corresponding whole protein lane image obtained from the same membrane.

### PASD Staining and LB Quantitation

Paraffin-embedded brain tissue was sectioned and stained using the periodic acid-Schiff diastase (PASD) method as described previously [19, 21]. Stained slides were scanned using the Hamamatsu Nanozoomer 2.0 HT digital slide scanner (40 × objectives), and the percentage area of hippocampus covered by LBs was quantified, as reported previously [24], using HistoQuant (3DHistech) by defining LBs signals based on pixel color. Values are expressed as % area.

### Quantification of Degradation-Resistant Glycogen

Aliquots of the frozen ground brain were left out to thaw at room temperature for 1 h to allow soluble glycogen to degrade, leaving behind degradation-resistant glycogen, as previously described [33]. The sample was then boiled in 30% KOH and glycogen precipitated in 70% ethanol with 57 mM sodium sulfate. Three further rounds of centrifugation and suspension in 67% ethanol with 15 mM LiCl followed, after which the pellet was resuspended in 100 mM sodium acetate pH 4.5 and digested with amyloglucosidase (Megazyme) in a 55 °C oven for 1 h along with digestion of blank controls. Glucose amount was determined in triplicate for each sample using a modified version of Lowry and Passonneau. Briefly, samples and glucose standards were mixed with 170 μL of G6PDH reaction mix (200 mM Tricine buffer/10 mM MgCl_2_ pH 8, 0.66 mM NADP; 1 mM ATP; 0.5 units G6PDH). Absorbance at 340 nm was acquired before (Abs1) and after (Abs2) addition of hexokinase (0.6 units in 4 μl of 200 mM Tricine/KOH, pH 8, 10 mM MgCl_2_). Abs2–Abs1 of glucose standards was used to derive glucose quantity in samples. Following normalization to fresh weight, glucose quantity represents degradation-resistant glycogen.

### Quantitative Real-Time PCR

RNA was extracted as described above. Immune system genes *Cxcl10*, *Ccl5*, *Lcn2*, and *C3* [5, 10] were quantified by quantitative real-time PCR (qRT-PCR) using the QuantStudio 7 Pro System thermo-cycler (ThermoFischer Scientific) and SYBR Green Master Mix (Bio-Rad Laboratories). Data are shown as fold change relative to control samples using the ΔΔC_q_ method with *Rpl4* as an internal control gene. Primers are listed in Table 1.

### Statistical Analysis

Data were analyzed and graphed using the GraphPad Prism software (v. 8.0.2; GraphPad Software). One-way ANOVA with Tukey’s post hoc test was performed for multiple comparisons. Student’s unpaired *t*-test was used to compare single means. For all comparisons, statistical significance was set at *p* < 0.05. Asterisks denote level of significance based on *p* value: *p < 0.05, **p < 0.01, ***p < 0.001, and ****p < 0.0001.

## Results

### Design and Validation of the Artificial miRNA Construct

The amiRNA contains a *Gys1*-targeting siRNA sequence embedded in an endogenous miRNA backbone (Fig. 1a). amiRNAs overcome a major shortcoming of earlier-generation short hairpin loop RNAs. The latter crowd out the complex that dices out their loops (DICER), leading to deficient processing of endogenous miRNAs and resultant toxicity. amiRNAs, on the other hand, by their natural miRNA sequences are pre-processed in a regulated fashion to occupy DICER in regular order without disturbing the processing of other miRNAs [34, 35].

We tested the functionality of multiple amiRNA constructs in cell lines. We transfected N2A cells and quantified *Gys1* mRNA levels using qRT-PCR and transduction efficiencies with GFP fluorescence and selected the most potent amiRNA for animal studies.

To confirm murine brain virus transduction and assess transduction pattern and distribution, we performed immunohistochemistry with an anti-GFP antibody. GFP expression was strong and broadly distributed across the brain in all three mouse models (Fig. 1c).

### AAV-amiRNA Decreases *Gys1* mRNA and Protein Levels

**Fig. 2:**
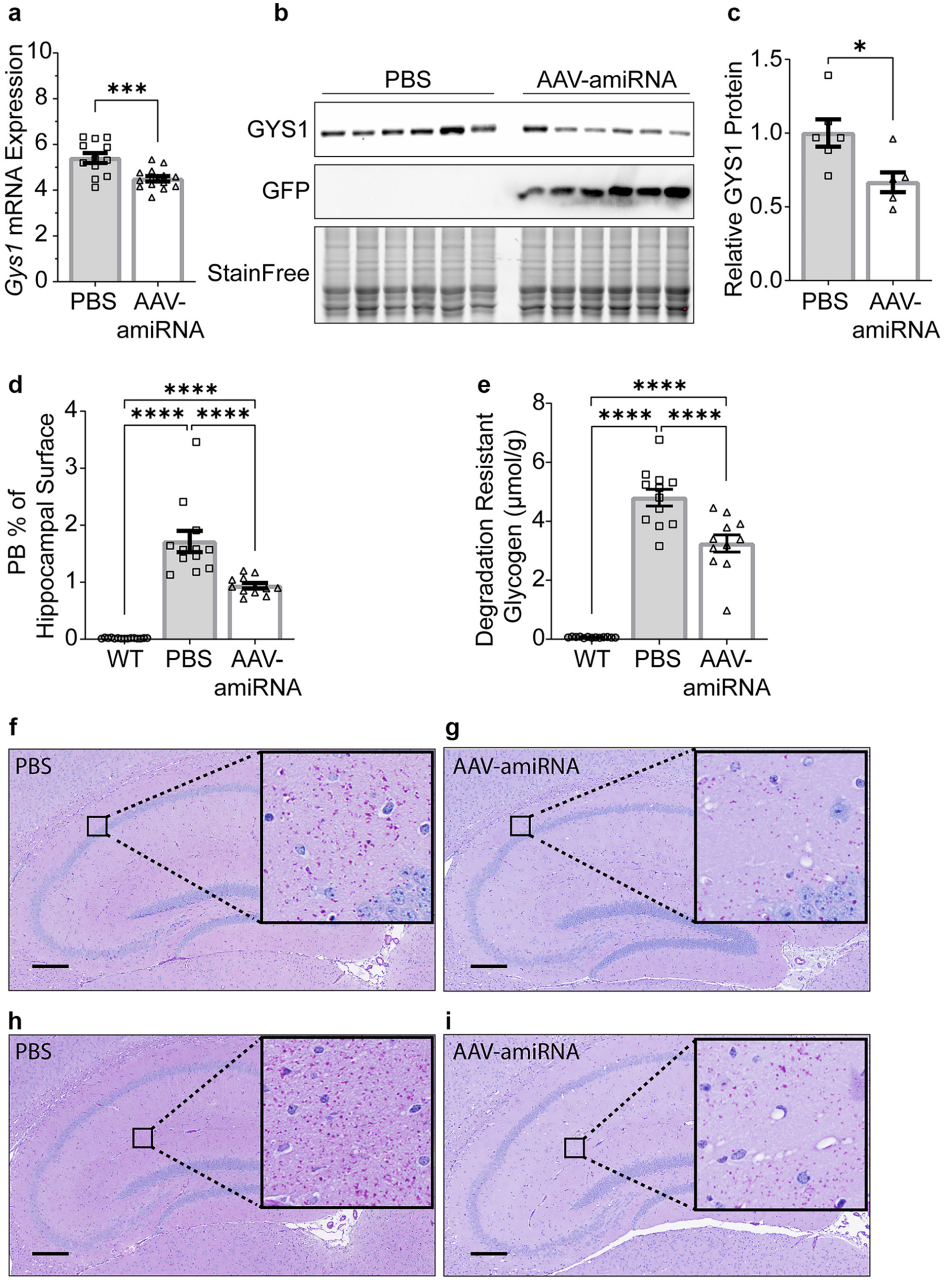
*Gys1* targeting AAV-amiRNA reduces *Gys1* mRNA and protein levels, insoluble glycogen, and PB accumulation in brains of the *Gbe1*^*Y239S*^ APBD mouse model. Neonatal mice (P2) were injected with PBS (*N* = 9–13 for each experiment) or AAV-amiRNA (*N* = 9–13 for each experiment), and WT mice (*N* = 9–13 for each experiment) were used as control. Mice were sacrificed at 3 months for brain tissue analysis. *Gys1* mRNA level (**a**) was measured by ddPCR. Representative brain GYS1 Western blots with stain-free gel as a loading control (**b**). Quantification of GYS1 Western blots normalized to stain-free gel shown in **c**. PB quantification in the hippocampus (**d**) and whole-brain degradation-resistant glycogen content (**e**). Representative micrographs of PASD-stained hippocampus of PBS (**f**, **h**) vs AAV-amiRNA (**g**, **i**) treated mice. Scale bar, 300 μm. Data are presented as mean ± SEM. Significance levels are indicated as *, *p* < 0.05; **, *p* < 0.01; ***, *p* < 0.001; ****, and *p* < 0.0001

**Fig. 3:**
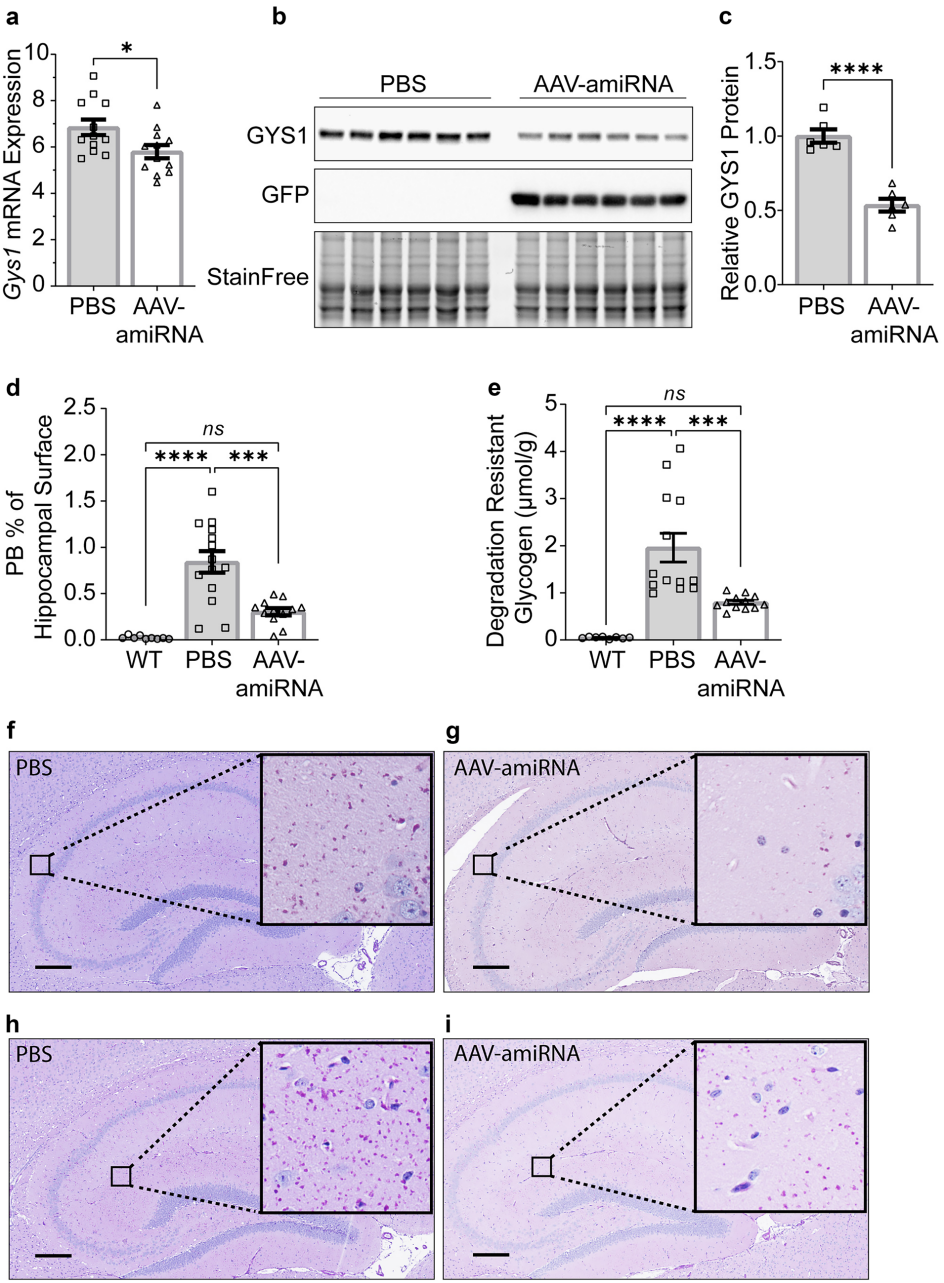
*Gys1* targeting AAV-amiRNA reduces *Gys1* mRNA and protein levels, insoluble glycogen, and PB accumulation in brains of the *Epm2a*^*−/−*^ LD mouse model. Neonatal mice (P2) were injected with PBS (*N* = 9–13 for each experiment) or AAV-amiRNA (*N* = 6 in WB quantification and *N* = 9–13 for other experiments), and WT mice (*N* = 9–13 for each experiment) were used as control. Mice were sacrificed at 3 months for brain tissue analysis. Gys1 mRNA level (**a**) was measured by ddPCR. Representative brain GYS1 Western blots with stain-free gel as a loading control (**b**). Quantification of GYS1 Western blots normalized to stain-free gel shown in **c**. PB quantification in the hippocampus (**d**) and whole-brain degradation-resistant glycogen content (**e**). Representative micrographs of PASD-stained hippocampus of PBS (**f**, **h**) vs AAV-amiRNA (**g**, **i**) treated mice. Scale bar, 300 μm. Data are presented as mean ± SEM. Significance levels are indicated as *, *p* < 0.05; **, *p* < 0.01; ***, *p* < 0.001; ****, and *p* < 0.0001

**Fig. 4:**
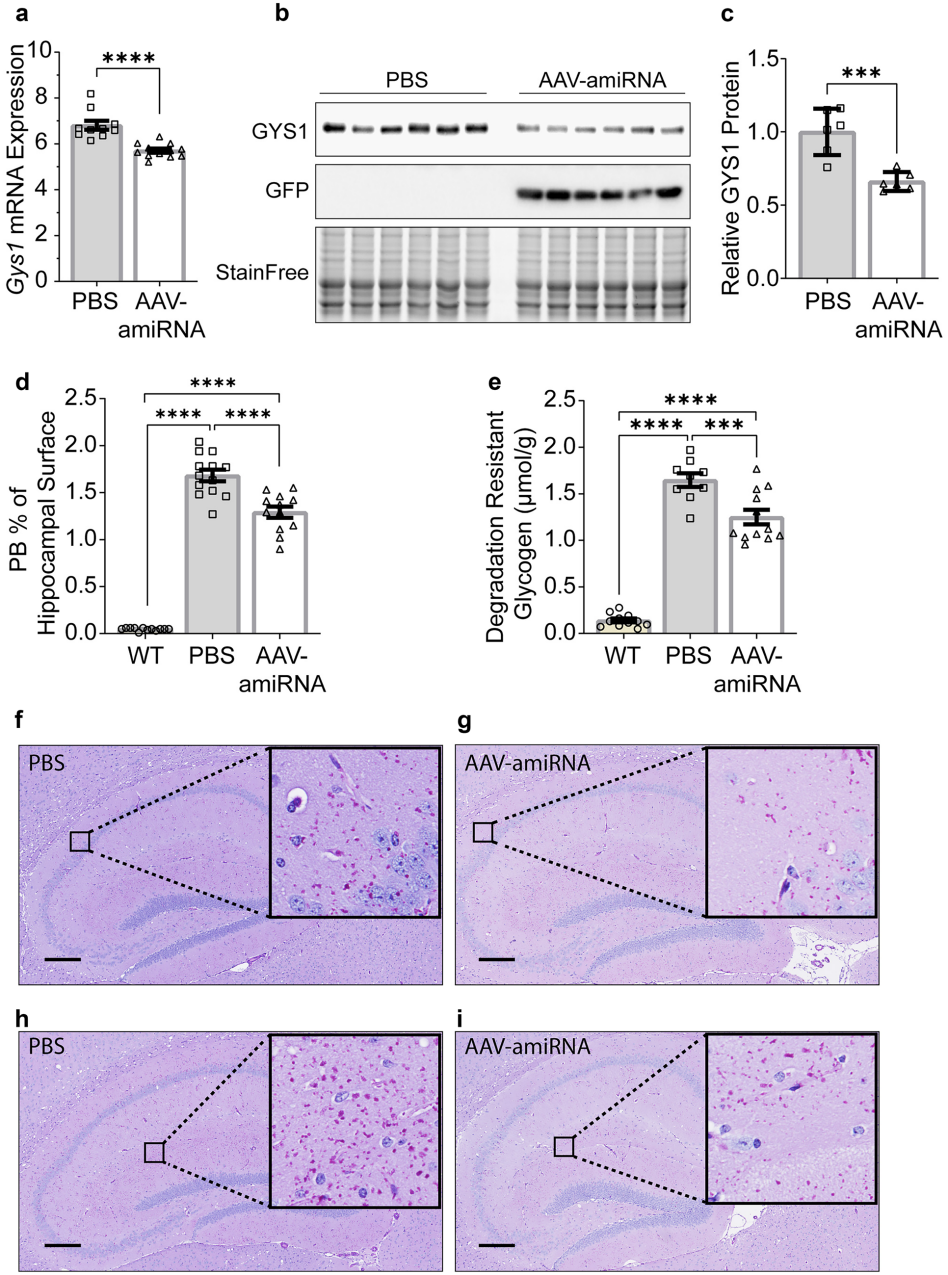
*Gys1* targeting AAV-amiRNA reduces *Gys1* mRNA and protein levels, insoluble glycogen, and PB accumulation in brains of the *Epm2b*^*−/−*^ LD mouse model. Neonatal mice (P2) were injected with PBS (*N* = 10 for each experiment) or AAV-amiRNA (*N* = 14 for each experiment), and WT mice (*N* = 9–13 for each experiment) were used as control. Mice were sacrificed at 3 months for brain tissue analysis. Gys1 mRNA level (**a**) was measured by ddPCR. Representative brain GYS1 Western blots with stain-free gel as a loading control (**b**). Quantification of GYS1 Western blots normalized to stain-free gel shown in **c**. PB quantification in the hippocampus (**d**) and degradation-resistant glycogen content (**e**). Representative micrographs of PASD-stained hippocampus of PBS (**f**, **h**) vs AAV-amiRNA (**g**, **i**) treated mice. Scale bar, 300 μm. Data are presented as mean ± SEM. Significance levels are indicated as *, *p* < 0.05; **, *p* < 0.01; ***, *p* < 0.001; ****, and *p* < 0.0001

To quantify the degree of *Gys1* mRNA knockdown in the brain, we used ddPCR on whole hemisphere extracts and found an approximately 15 to 17% reduction in the AAV-amiRNA-treated mice of all three genotypes (Figs. 2a, 3a, and 4a). We then performed quantitative Western blotting on the same extracts and found a larger, 33 to 46%, reduction of GYS1 protein (Figs. 2b, c, 3b, c, and 4b, c).

### AAV-amiRNA Reduces PBs and Degradation-Resistant Glycogen

At 3 months of age, PBs are clearly visible throughout the brain in all three disease models, and changes in PB amounts can be quantified histologically [5, 24]. To measure the effect of AAV-amiRNA on PBs, we stained sections from paraffin-embedded hemispheres with PASD and quantified % PASD signal per hippocampal area. AAV-amiRNA treated mice showed 23 to 64% reduced PB accumulations in the hippocampus compared to vehicle-treated mice (Figs. 2d, f–i, 3d, f–i, and 4d, f–i).

Brain glycogen in APBD and LD consists of normal water-soluble glycogen and precipitated and aggregated glycogen (polyglucosans, PBs) [36–38]. The soluble portion degrades at room temperature, while the insoluble does not. Biochemical quantification of the latter is a second standard way to measure PB load [20]. AAV-amiRNA-treated mice had 24 to 60% reduced degradation-resistant glycogen in whole-hemisphere extracts compared to vehicle-treated mice (Figs. 2e, 3e, and 4e), comparable to the PB reductions as measured histochemically.

### AAV-amiRNA Ameliorates PB-Associated Immune Activation

The LD mouse models are full knockouts of the *Epm2a* or *Epm2b* genes. They recapitulate the neuropathology of LD. The PBs appear first and are well established by 3 months of age. At 16 months, there is astrogliosis and microgliosis, and transcriptomic studies show that 94% of 229 misregulated mRNAs are immune or inflammatory pathway gene transcripts [10]. At this age, there are behavioral deficits, myoclonus, seizures, and seizure susceptibility to proconvulsant drugs [29, 30]. Importantly, all these abnormalities are prevented or improved with genetic downregulation of glycogen synthase and resultant inhibition of LB formation [5, 18, 20, 21, 24, 39–41]. Of the 229 genes misregulated at 16 months, a subset, *Cxcl10*, *Ccl5*, *Lcn2*, and *C3*, were reported to already be upregulated at 3 months of age [5, 10, 24].

The APBD mouse model has the most common human APBD mutation, Tyr239Ser [31]. It exhibits larger amounts of PBs than LD by 3 months of age, and by 8 months astrogliosis, microgliosis, and behavioral and motor deficits, all of which are preventable by glycogen synthase downregulation [19]. Transcriptomics have not been carried out, but three (*Cxcl10*, *Lcn2*, and *C3*) of the above four neuroimmune genes are upregulated at 3 months of age.

**Fig. 5:**
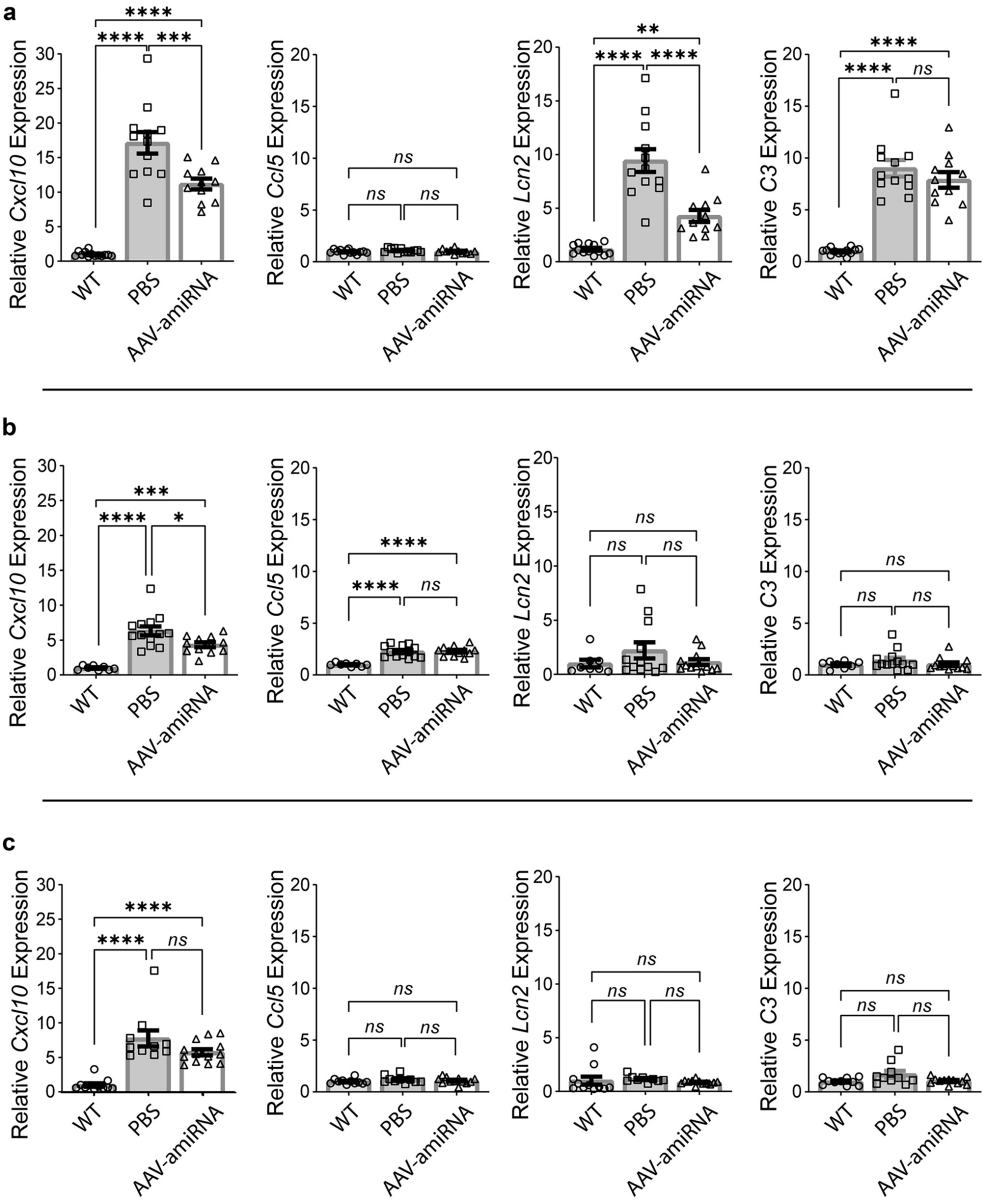
AAV-amiRNA ameliorates PB-associated immune activation. Neonatal mice (P2) were injected with PBS or AAV-amiRNA and sacrificed at 3 months for brain tissue analysis. WT indicates the wild-type control group. For each panel, from left to right, *Cxcl10*, *Ccl5*, *Lcn2*, and *C3* were used as neuroinflammation markers, and relative mRNA expression levels were analyzed by qRT-PCR for *Gbe1*^*Y239S*^ (**a**), *Epm2a*^*−/−*^ (**b**), and *Epm2b*^*−/−*^ (**c**) mice. In panel **a**: WT (*N* = 13), PBS (*N* = 12), and AAV-amiRNA (*N* = 11). In panel **b**: WT (*N* = 8), PBS (*N* = 13), and AAV-amiRNA (*N* = 12). In panel **c**: WT (*N* = 11), PBS (*N* = 10), and AAV-amiRNA (*N* = 13). Data are presented as mean ± SEM. Significance levels are indicated as *, *p* < 0.05; **, *p* < 0.01; ***, *p* < 0.001, ****, and *p* < 0.0001

We quantified the expressions of *Cxcl10*, *Ccl5*, *Lcn2*, and *C3* in wild-type and AAV-amiRNA or vehicle-treated APBD and LD mice. We confirmed the upregulation of *Cxcl10*, *Lcn2*, and *C3* in the APBD mice. Expressions of two of these genes, *Cxcl10* and *Lcn2*, significantly reduced with the *Gys1*-targeting AAV-amiRNA. Levels of *C3* also decreased though not to statistical significance (Fig. 5a). In the *Epm2a*^*−/−*^ mice, two genes, *Cxcl10* and *Ccl5*, were upregulated, and the level of *Cxcl10* significantly corrected with the treatment (Fig. 5b). In the *Epm2b*^*−/−*^ mice, one gene, *Cxcl10*, was upregulated. This reduced with the treatment but not to statistical significance (Fig. 5c).

## Discussion

Downregulating glycogen synthase has emerged as a leading path to treating APBD and LD. Transgenic and CRISPR/Cas9-based knockout and knockdown experiments [5, 16–23] laid the groundwork for the development of an ASO-based therapy presently being developed [24] and small molecule inhibitors of the glycogen synthase enzyme as potential oral drugs [42]. The main drawback for an ASO therapy is that it needs to be administered through repeat lumbar punctures multiples times a year lifelong. Challenges that face small molecules include that they do not immediately have the specificities of gene-based therapies and, as in this case, require further development to allow blood–brain barrier passage. In the present work, we establish proof of principle for a gene therapy-based GYS1-directed single lifetime dose treatment for APBD and LD. While the same could potentially be achieved with CRISPR/Cas9 technology, the latter faces a longer runway to the human translation given unresolved concerns with Cas9 immunogenicity and potential off-target activity.

The core sequence of the amiRNA studied here and that of the ASO studied previously [24] target murine *Gys1* RNA. Safe and effective oligonucleotides that downregulate human *GYS1* are in development. An important observation from the present study relevant to this is the disparity between the level of *Gys1* mRNA reduction (13–18%) and the reductions of GYS1 protein, abnormal glycogen, and PBs (23–64%) (Figs. 2, 3, 4, and 5). This same disparity was noted in two other recent studies where *Gys1* was downregulated by other means [5, 21]. Disconnection between expression and translation is not common but certainly not unknown [43]. The practical implication here is that the level of GYS1 protein appears to be exquisitely responsive to changes in the level of its mRNA. If confirmed in human cell lines and humanized (*Gys1* replaced by *GYS1*) APBD and LD mouse models, this represents an important advantage. Selection of the human ASO or amiRNA sequence would not be restricted to the few particularly potent options, but can be selected from a wider range of possible oligonucleotides. Secondly, comparatively lower doses of ASO or viral genome counts of AAV-amiRNA would be required, and in the case of the latter, there will be options in selecting among promoters with different strengths and other characteristics.

The next steps in the translation of our results to a clinical trial include the following. The work needs to be repeated in human cell lines with a new amiRNA that specifically targets primate (human and nonhuman) *GYS1* sequence and demonstrates that a degree of GYS1 downregulation similar to the results obtained in the present study can be achieved. The new amiRNA should then be administered to nonhuman primates by ICV (or other intra-CSF) injection in different doses to establish an effective dose that results in a similar GYS1 downregulation as in the present study. The latter experiment would also serve in monitoring for any signs of toxicity including neuroinflammation or neurodegeneration. Finally, the eventual clinical trials could be controlled with a sham-injection arm or against the results of published or presently ongoing LD and APBD natural history studies [44–46].

In recent years, additional PB diseases have come to light including GYG1 deficiency [47], RBCK1 deficiency [48], and KLHL24 deficiency [49]. The first two are skeletal and cardiac myopathies and the third a cardiomyopathy, the last two fatal from cardiac disease. GYG1 is the glycogen synthesis initiator protein. RBCK1 [50] and KLHL24 [51] are components of protein complexes involved in ubiquitination. How the latter two connect with glycogen metabolism is not known, although tantalizing very recent results suggest that RBCK1 may ubiquitinate glycogen itself [52]. In any event, the abnormal glycogen in these diseases all but certainly has overextended branches driving it to precipitation, and glycogen synthase downregulation should mitigate the problem as in LD and APBD. In fact, our recent results already confirm this for RBCK1 deficiency [53, 54]. An important point to note is that the present GYS1 downregulating therapy or others are expected to slow or halt PB formation but not remove existing ones. This was confirmed recently in our *Gys1* conditional knockout work, where the gene was deleted in LD mice in the mid-course of the disease [21]. As such, these therapies would benefit newly diagnosed patients most, preventing them from progressing beyond early symptoms. They would be expected to benefit patients with the more advanced disease only in preventing further progression.

The basis of downregulating glycogen synthase for PB diseases is to rebalance the activities of glycogen synthase and branching enzyme and thus prevent the former from outpacing the latter and forming overlong glycogen branches. However, glycogen synthase downregulating therapies could also benefit others of the more than 15 glycogen storage diseases where normally structured (branched) glycogen accumulates in abnormally large amounts. By far, the most common of these glycogenoses is Pompe disease caused by acid maltase deficiency and accumulation of normally branched glycogen in lysosomes. Results to date with a *Gys1* targeting ASO are positive [55].

In summary, we have shown that a virally delivered glycogen synthase downregulating miRNA effectively reduces the pathogenic PBs in the prototypical PB diseases APBD and LD, along with improvements of their early neuroimmune abnormalities. This work suggests that a single lifetime dose treatment for these fatal neurological diseases is possible. Glycogen synthase is the effector enzyme not only for glycogen branch lengths but also glycogen synthesis in general. Results to date suggest that a single drug acting on this enzyme may benefit a range of severe and common diseases of both polyglucosan and normal glycogen accumulation.

## Supplementary Information

Below is the link to the electronic supplementary material.

Supplementary file1 (PDF 1225 KB)

Supplementary file2 (PDF 1225 KB)

Supplementary file3 (PDF 1225 KB)

Supplementary file4 (PDF 1225 KB)

Supplementary file5 (PDF 1225 KB)

Supplementary file6 (PDF 1225 KB)

Supplementary file7 (PDF 1225 KB)
